# Chromatin‐transcription interface: The secret of eternal youth?

**DOI:** 10.1111/acel.13927

**Published:** 2023-07-10

**Authors:** José M. Izquierdo

**Affiliations:** ^1^ Centro de Biología Molecular Severo Ochoa Consejo Superior de Investigaciones Científicas‐Universidad Autónoma de Madrid (CSIC‐UAM) Madrid Spain

**Keywords:** aging, chromatin structure, healthspan, lifespan, longevity, transcriptional elongation speed

## Abstract

In their recent study in Nature, Debès et al. report an increase in RNA polymerase II (Pol II)‐mediated transcriptional elongation speed associated with chromatin remodeling during aging in four metazoan animals, two human cell lines, and human blood. Their findings might help us understand why we age through evolutionarily conserved essential processes, and open a window to the molecular and physiological mechanisms influencing healthspan, lifespan and/or longevity.

Aging is the progressive breakdown of physiological homeostasis throughout life, and is a major risk factor for numerous chronic diseases. Not surprisingly, age‐related declines in health carry a significant economic and clinical burden (Cylus et al., 2019). Indeed, the increase in the elderly population represents a global public health challenge and, unfortunately, we are ill prepared for the physical, mental, social, and health consequences that this entails. There is thus is a pressing need to understand how aging is influenced by lifestyle choices and by environmental and socio‐economic factors (Cylus et al., 2019), and to develop better approaches to study the biology of aging and to quantify age‐dependent changes in physio(patho)logical functions (López‐Otín et al., 2023).

Aging‐associated cellular and molecular loss‐of‐functions are interconnected to at least 12 hallmarks: genomic instability, telomere attrition, epigenetic alterations, loss of proteostasis, disabled macroautophagy, deregulated nutrient‐sensing, mitochondrial dysfunction, cellular senescence, stem cell exhaustion, altered intercellular communication, chronic inflammation, and dysbiosis. These hallmarks are grouped into three categories—primary, antagonistic and integrative—and are linked to others such as organizational features of spatial compartmentalization, maintenance of homeostasis, and adequate responses to stress (López‐Otín et al., 2023). Whether a hierarchy exists among the primary aging hallmarks that could order this complex multifactorial phenomenon with the goal of slowing or even reversing aging is an emerging issue. In this quest, Debès and colleagues report a new hierarchical and regulatory dimension associated with aging involving increased transcriptional elongation speed bound to modifications of transcript structure and chromatin organization/structure as an evolutionarily conserved mechanism (Debès et al., 2023).

The fascinating article by Debès et al. addresses the aforementioned aging‐associated physio(patho)logical scenario by investigating the kinetics of transcription during aging in five metazoan species: nematodes (*C. elegans*), fruitflies (*D. melanogaster*), mice (*M. musculus*), rats (*R. novergicus*) and humans (*H. sapiens*). They measured Pol II‐mediated transcriptional elongation speed using high‐throughput transcriptome profiling in different adult tissues and stages (Figure 1), observing an increase in elongation speed with age in all species and tissue types examined in an intron length‐ and gene position‐independent manner. This was confirmed by monitoring transcription kinetics in proliferating (young) and senescent human IMR‐90 cells using 4‐thiouridine labeling of nascent RNA. To establish whether Pol II‐associated elongation speed was affected by lifespan‐extending conditions, they analyzed two well‐established interventions that cause longevity: inhibition of insulin‐IGF signaling in *C. elegans* and *D. melanogaste*r, and caloric restriction in wild type and *Irs*‐null mice (Chen et al., 1996; Rogalski et al., 1988; Selman et al., 2008). In most cases, lifespan‐extending conditions resulted in a significant reduction of Pol ll velocity (Figure 1).

**FIGURE 1:**
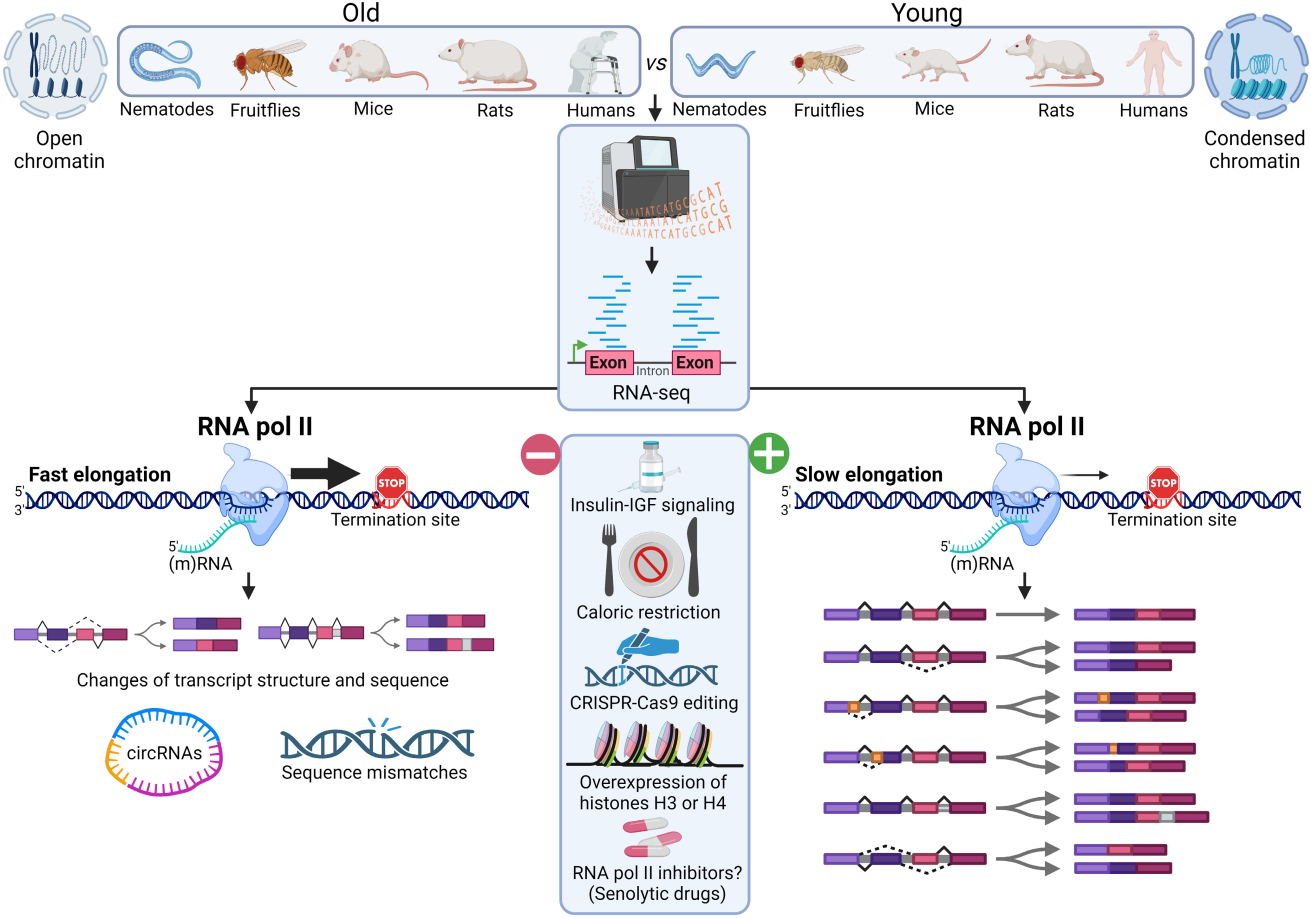
Chromatin organization and RNA Pol II transcription elongation speed are evolutionarily conserved regulatory mechanisms during aging in metazoans. High‐density transcriptomic analyses from total and nascent RNA transcript tagging in five metazoan species (*C. elegans, D. melanogaster*, *M. musculus*, *R. novergicus* and *H. sapiens*) during their transition from young to aged adults reveals a significant increase in Pol II transcriptional elongation speed during aging. The Pol II‐dependent increase in elongation speed is accompanied by an increase in the efficiency of alternative splicing, generation of circular RNAs, and sequence mismatches. Using specific mutants associated with Pol II activity, the process could be reversed in the context of insulin‐IGF signaling inhibition in fruitflies and nematodes and caloric restriction in mice, which are both known to increase longevity. The animals lived 10%–20% longer than their non‐mutant controls. When the mutation was reversed using CRISPR‐Cas9 editing, the animal lifespan was shortened, indicating a strong casual link between Pol Il elongation speed and lifespan extension. In the same vein, using human cell lineages, the cellular senescence state was reversed upon overexpression of histone H3 or H4. This result correlates with the fact that an increase in transcriptional elongation rate in aged animals and human cells corresponds to an open chromatin state or euchromatin, whereas a condensed chromatin state or heterochromatin is observed in young animals. Whether Pol II inhibitors can act as senolytic drugs by reducing Pol II‐dependent transcriptional elongation rates without toxicity and be accompanied by a rejuvenation phenotype, or increased lifespan and longevity, remains to be investigated. Pol II, RNA polymerase II. This figure was created with BioRender.com

To determine the underlying mechanisms involved in this process, the authors performed enrichment analysis on the 200 genes showing the highest increase in Pol ll speed during aging in worms, fly brain, mouse kidney and liver, and rat liver. However, only some genes involved in metabolic activity were slightly enriched in three or more species, indicating that no selective cellular processes were consistently affected across species and tissues. The authors next sought to test whether changes in Pol lI elongation speed are causally involved in aging using genetically‐modified worm and fly strains with point mutations in a major Pol II subunit that reduces elongation speed. Results showed that slowing down Pol II increased the lifespan of *C. elegans* and *D. melanogaster* by 20% and 10%, respectively. This was confirmed using CRISPR‐Cas9 editing to repair the point mutant in *C. elegans*, which restored the lifespan to wild type levels (Figure 1).

Splicing is a co‐transcriptional regulatory event (Bentley, 2014), and there is robust evidence for a link between chromatin organization and both transcriptional rate (Muniz et al., 2021) and constitutive and alternative splicing control (Naftelberg et al., 2015; Tilgner et al., 2009). Debès et al. next examined datasets from total and nascent RNA sequencing using splicing efficiency criteria, observing an increase of spliced over unspliced exon junctions during aging, and the opposite under lifespan‐extending conditions. Faster transcription rates reduce the time for splicing choices, which increases the frequency of anomalous/erroneous splicing events associated with aging and shortened lifespan (Rogalska et al., 2023). However, most databases have been created using young or embryonic tissues. To search for erroneous splicing linked to rare isoforms during aging, Debès et al. used the Leafcutter tool, which performs de novo quantification of exon‐exon junctions based on split‐mapped RNA‐sequencing reads (Li et al., 2018). They detected rare exon‐exon junctions often resulting from exon skipping or from the usage of cryptic splice sites, allowing them to conclude that the average fraction of rare splicing events increases during aging solely in worms and flies, and that these events are reverted by lifespan‐extending interventions. The authors also found the enhanced formation of circular RNAs (circRNAs) (Zhang et al., 2016), which have previously been associated with increased Pol II velocity; however, they observed either increased or unchanged levels of circRNAs during aging. Faster Pol II elongation correlates with a general increase in the levels of circRNAs and can be a consequence of the overall reduced quality in RNA production. To explore the idea that faster Pol II speeds increase other types of transcriptional errors, Debès et al. measured the number of mismatches in aligned reads for each gene, excluding genomic variations and other artefacts, observing that the average fraction of mismatches increased with age, but decreased under lifespan‐expanding treatments. Likewise, slow Pol II mutants exhibited reduced numbers of mismatches compared with controls in three out of the four comparisons (Figure 1).

Given that nucleosome positioning along DNA is related to Pol II transcriptional elongation speed and splicing regulation, and that nucleosomal density in chromatin is reduced in aged eukaryotic cells (Oberdoerffer, 2010), the authors next evaluated alterations in chromatin structure in early (proliferating) and late (senescent)‐passage IMR‐90 cells. Results indicated that the transition from a proliferative state to replicative senescence was associated with small but significant changes in chromatin structure, suggesting that nucleosome density and positioning changes are affected by Pol II elongation. Additionally, they showed that overexpression of histone H3 or H4 decreases Pol II elongation speed in IMR‐90 cells and compensated for the aging‐induced loss of core histones to restrict senescence entry. To address the role of nucleosome density in organismal lifespan, the authors overexpressed H3 in *Drosophila* glial cells, which increased lifespan. However, previous findings had already demonstrated that chromatin structure modulates Pol II elongation speed and lifespan in yeast, *C. elegans* and *D. melanogaster* (Feser et al., 2010; Lu et al., 2021; Sural et al., 2020) (Figure 1). Taken together, the observations of Debès et al. point to an evolutionarily conserved role of aging mechanisms associated with the chromatin‐transcription regulatory axis, likely because these complex cellular machineries are highly interconnected and conserved from invertebrates to vertebrates.

Throughout this *perspective*, I have detailed the most important discoveries of Debès et al., and the mechanistic implications linking the chromatin‐transcription interface to aging‐related cellular and physio(patho)logical processes; however, there are several concepts that merit further study (Figure 2). For instance, what are the molecular implications of increased Pol II speed on other post‐transcriptionally regulated processes, such as RNA stability/turnover, control of noncoding (nc) RNAs (both large and short), and protein degradation and mRNA translation rates? Historically, research in this field focused on transcriptional initiation, which established its essential role in the regulation of gene expression. Over the years, however, it has become evident that other transcriptional steps are also critical in controlling gene expression. Mutants of RNA Pol II with different transcription speeds have provided important advances in our understanding of its essential role in many co‐transcriptional processes. RNA Pol II speed or elongation rate—the number of nucleotides synthesized per unit time is an important determinant of transcriptome composition, as it also controls splicing, polyadenylation and transcription termination, thus regulating the production of alternative splice variants, circRNAs, long non‐coding RNAs (lncRNAs), ncRNAs, small RNAs (miRNAs), alternatively polyadenylated transcripts, or even the length final of transcripts. The heterogeneity of RNA populations and their inter‐ and intrainteractions that can alter RNA availability and half‐life can impact protein homeostasis or proteostasis, promoting changes in global and specific rates of de novo translation, and in the dynamics of protein degradation. The rate of RNA Pol II itself is regulated in response to intra‐ and extracellular stimuli and may, in turn, affect the composition of the transcriptome. The available evidence points to a potentially important role of transcriptome composition through regulation of RNA Pol II velocity in adapting cells to changing environments, meaning that RNA Pol II elongation speed may be fundamental for the regulation of pathophysiological processes across development and aging by RNA heterogeneity‐dependent dynamics, influencing de novo protein synthesis and stability/turnover (Muniz et al., 2021). Further, these essential cell machineries are biological clocks with regulatory instructions for the spatiotemporal expression of genes during development, including growth, cell homeostasis, senescence and aging (Panigrahi & O'Malley, 2021) (Figure 2).

**FIGURE 2:**
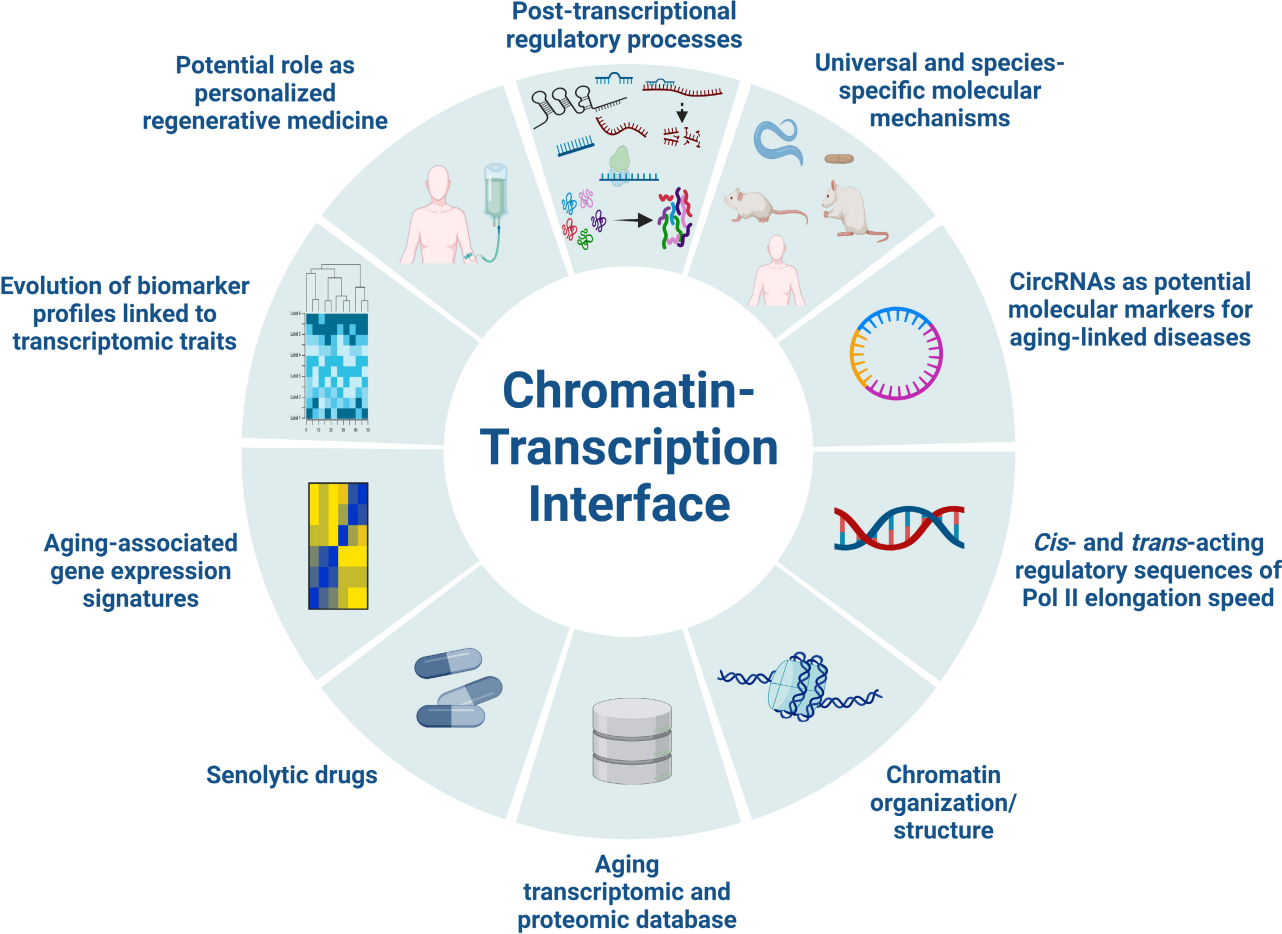
Future challenges of chromatin‐transcription interface. Mechanistic, prognostic and therapeutic aspects to be investigated related to the chromatin‐transcription regulatory axis and associated with aging and its pathologies. This figure was created with BioRender.com.

Another feature that remains poorly studied is whether there is a combination of universal and species‐specific molecular mechanisms governing the manifestation of aging‐associated characteristics that influence lifespan control. Given the evolutionary conservation of some aging‐linked gene regulatory networks, a combination of universal and species‐specific molecular mechanisms might be expected to exist in all multicellular organisms according to a recent report (Tyshkovskiy et al., 2023). This notion has been also tested in another recent exhaustive analysis of the molecular and genetic mechanisms underlying age‐related decline in flies through the so‐called “Cellular Atlas of Aged Flies”, demonstrating the existence of transcriptomic variations during aging for a number of expressed genes, with different cell types differentially affected by various features of aging (Lu et al., 2023). Likely, synergistic or additive effects of selected interventions trigger a spectrum of responses at the gene expression level in many aging‐associated hallmarks, leading to a delay/increase in aging and an increase/delay in lifespan (López‐Otín et al., 2023) (Figure 2).

Another challenging topic is related to whether circRNAs could be considered as potential molecular markers for aging‐related disorders. What we know so far suggests that RNA Pol II speed, through controlling splicing efficiency, is also implicated in the regulation of circRNA production. CircRNAs are produced by back‐splicing of pre‐mRNAs, specifically, the joining of a downstream 5′ splice site to an upstream 3′ splice site in the reverse orientation, forming a covalently closed loop. Several studies have reported emerging functional roles of circRNAs through their ability to regulate gene expression at multiple levels and to contribute to transcriptome diversity. For example, circRNAs can function as competitive endogenous RNAs by binding to miRNAs through miRNA response elements (i.e., “miRNA sponges”), thereby reducing the quantity of miRNAs available to target mRNA, and subsequently promoting mRNA stability or protein expression. Tens of thousands of circRNAs have been identified in metazoans, and some are evolutionarily conserved. Notably, there is accumulating evidence for the involvement of circRNAs in the regulation of age‐related pathologies, including cardio‐cerebrovascular disease, neurodegenerative disease, cancer, diabetes, rheumatoid arthritis, and osteoporosis (Pan et al., 2020). The association of circRNAs with age‐related pathologies highlights their potential as diagnostic biomarkers and therapeutic targets for disease management (Pan et al., 2020) (Figure 2).

Another interesting question is whether there are *cis*‐ (specific genomic sequences) and/or *trans*‐ (splicing compounds) regulatory elements that modulate (either enhance or repress) Pol II elongation speed during aging (Mimoso & Adelman, 2023) (Figure 2). In this regard, RNA Pol II speed can be controlled by different factors including, but not restricted to, DNA sequence, gene structure, histone modifications, chromatin remodelers, and RNA Pol II‐associated factors and modifications (Muniz et al., 2021). For instance, eukaryotic transcription units are also characterized by conserved 5′ to 3′ profiles of specific histone modifications deposited co‐transcriptionally. Histone modifications profiles are sensitive to RNA Pol II speed (Soares et al., 2017). In the same vein, the carboxy C‐terminal domain (CTD) of RNA Pol II, which consists of tandem repeats of the heptapeptide YSPTSPS, plays a central role in the coordination of co‐transcriptional processing through its association with a large number of enzymes and protein/RNA‐binding factors. The CTD is the target of many post‐translational modifications, yielding specific patterns (referred to as “the CTD code”) that are recognized by factors with essential roles throughout the transcription cycle. Thus, gene modifications, remodelers, and signaling pathways could contribute to aging in different cell types (Muniz et al., 2021) (Figure 2).

Another exciting issue is whether aging follows a directional process where chromatin structure, its organization, and Pol II elongation speed, act as primary events establishing a hierarchical order for other aging‐related traits and hallmarks (Figure 2). Local chromatin architecture is an important regulator of transcription, and regulation of gene expression is linked to the organization of the genome. Age‐related chromatin alterations occur at all levels of genome organization (transitions from heterochromatin i.e., condensed to euchromatin i.e., open), accompanied by changes in gene expression profiles. A deeper understanding of the epigenetic mechanisms might reveal critical links between gene expression regulation and multiple cellular biological processes (i.e., primary aging‐associated hallmarks) that can be exploited to promote youthful states in cells and organisms. Dissecting the dynamic interplay between these biological pathways and epigenetic parameters in adult cells, tissues and organisms that have been subjected to rejuvenation strategies will broaden our understanding of how to make our bodies younger with minimal side effects (Zhang et al., 2020). In this respect, Debès et al. establish a functional connection between aging, chromatin accessibility, and transcriptional regulation (Figure 2).

Another question that remains open is, are databases containing transcriptomic information (including splicing variants and ncRNAs) and proteomic data (comprising protein isoforms and their interactomes) from aged cell and animal models, encompassing both sexes, comprehensive (Figure 2)? Future gene expression profiling should include full transcript analysis of the isoforms and stoichiometry of mRNAs, lncRNAs and small RNAs in senescent cells and adult animal models at different stages of proliferation, differentiation, and in various physiological and aging states, learning and stress conditions. These efforts should be complemented by cell‐ and organoid‐based and in vivo studies using strategies for conditional and tissue‐specific or cell type‐specific gain‐ and loss‐of‐function of RNAs. More broadly, identifying and understanding the roles of RNA regulatory networks in multicellular aging will require the determination of the interplay between chromatin modifications, RNAs, proteins and the genome in the assembly of the nuclear domains essential for chromatin organization, enhancer function, transcription and splicing. In this regard, the power of machine learning must be harnessed to interrogate large genomic, epigenomic, transcriptomic, proteomic and phenomic datasets to identify causal links and pathways during aging processes (Tyshkovskiy et al., 2023) (Figure 2).

Senolytic drugs were developed to ablate senescent cells and to reverse age‐related phenotypes in a cell type‐specific manner. Thus, could inhibitors targeting Pol II and its machinery be considered as novel senolytic drugs (Figure 2)? Changes in the regulation of gene transcription by Pol II underlie virtually every complex human disease. Inhibitors targeting the general Pol II transcription machinery are now candidate therapeutics in cancer and other complex conditions including aging‐associated diseases (diabetes, neurological disorders, immune disorders, and cardiovascular disease). Pol II‐associated inhibitors include several potent natural toxins, such as α‐amanitin, which inhibits RNA pol II, and actinomycin D (ActD), commonly used as an experimental tool to inhibit transcription. ActD was originally developed as an antibiotic and was the first drug of this class to have therapeutic benefit in cancer. However, ActD also has inhibitory capacity on RNA pol I > RNA pol II > RNA pol III, and has been employed in four phase II and six phase III cancer trials as a single‐agent or in combination. Furthermore, cyclin‐dependent kinase (CDK) inhibitors affect Pol II transcription (e.g., flavopiridol, roscovitine, SNS‐032, and dinaciclib), although their clinical utility has been limited by their toxicity. Recently, more selective inhibitors for individual CDKs have been developed (e.g., THZ1 for CDK7, cortistatin A for CDK8, BAY 1143572 for CDK9, and THZ531 for CDK12 and CDK13) (Martin et al., 2020). Perhaps the next generation of Pol II inhibitors will be useful in the development of novel anti‐aging approaches (Wouters et al., 2017). Interestingly, a very recent study reported that taurine, a quasi‐essential nutrient found in all eukaryotic organisms and highly expressed in mammalian tissues (and also a common ingredient in health supplements and energy drinks) reduced cellular senescence, protected against telomerase deficiency, suppressed mitochondrial dysfunction, decreased DNA damage, attenuated inflammation and delayed aging in several models (Singh et al., 2023). Another positive benefit of taurine included evident epigenetic changes in DNA and histone methylation, which could alter chromatin conformation/structure, affecting transcription and modulating Pol II elongation velocity. Future comprehensive studies may shed light on the differences in the mechanisms of longevity interventions operating via anti‐aging or aging‐independent routes. (Figure 2).

The comprehensive analysis of gene expression across species and identification of longevity biomarkers might be key to discovering novel senolytic and/or geroprotective drugs (Figure 2). For example, a recent study addressed the characterization of mammalian longevity gene expression signatures in 41 species, including the long‐lived naked mole rat, Brandt's bat, and the bowhead whale (Tyshkovskiy et al., 2023). The authors identified species‐specific, tissue‐specific, and universal transcriptomic biomarkers of mammalian aging through a quantitative meta‐analysis of 92 publicly available datasets, and demonstrated that the identified signatures can be used to discover and characterize regulatory interventions of longevity in mammals. Thus, establishing the shared and distinct molecular mechanisms of longevity, as well as their causal relationship to aging, is of critical importance for understanding the drivers of longevity and for the development of effective geroprotectors. The authors identified biomarkers and longevity interventions, including KU0063794, an mTORC1/2 inhibitor, which prolonged life expectancy and health in mice. Overall, this study uncovered universal and distinct strategies of longevity regulation within and between species, providing tools for discovering longevity interventions (Tyshkovskiy et al., 2023) (Figure 2).

Can the evolution of biomarker profiles associated with transcriptomic signatures of longevity be established as indicators of our healthspan expectancy and future therapeutic interventions (Figure 2)? This is the conclusion derived from the "Aging Fly Cell Atlas,” a single‐nucleus transcriptomic map of aging in *Drosophila* (Lu et al., 2023). This study characterized 163 distinct cell types through the in‐depth analysis of changes in tissue cell composition, gene expression, and cell identities. In addition, the authors developed aging clock models to predict fly age, showing that ribosomal gene expression is a conserved predictive factor for age. Combining all aging features, they found distinctive cell type‐specific aging patterns. This atlas provides a valuable resource for studying fundamental principles of aging in complex organisms, and may provide new insights into an evolutionarily conserved phenomenon and the potential roles of multinucleated cells in age‐related diseases. Future transcriptomic signatures of longevity may be used to identify lifespan‐ and healthspan‐extending interventions based on gene expression profiles, thus facilitating the discovery of novel geroprotectors (Figure 2).

The ultimate and most important goal of these studies should be question whether there are realistic possibilities of extrapolating these and other findings to personalized regenerative medicine (e.g., muscle disorders, neurological diseases, inflammatory pathologies, and aging) and to other aging‐associated pathologies. The promise of regenerative medicine to revolutionize health duration, life expectancy and/or longevity beyond the laboratory will be some time in coming, although the first steps have already been taken (Figure 2). In the example of taurine, however, the doses used in animal studies (Sing et al., 2023) are equivalent to 3–6 g of taurine for an 80‐kg body‐weight individual, and it is not recommended that people consume this over‐the‐counter supplement in an attempt to preserve health or slow aging. Nevertheless, taurine represents an opportunity to develop large‐scale clinical trials in humans with different age groups as a “source” of life/health/youth that rejuvenates and extends our healthy life expectancy delaying the onset of aging‐associated diseases and even aging itself (Singh et al., 2023). The potential advantages of taurine include its natural occurrence in our body and its availability from the diet. It also has no known toxic effects, it can be enhanced by exercise, and, as a naturally occurring biomolecule, it cannot be exploited through a commercial patent.

In summary, Debès et al. establish the evolutionary conserved role of Pol II elongation speed associated with aging‐linked changes in chromatin structure in five separate metazoan species as early molecular mechanisms contributing to aging. These findings potentially define how to slow aging and expand healthspan. Future comprehensive studies will shed light on the synergism and/or prevalence among the aging‐associated specific and universal molecular mechanisms across species and multi‐tissular organisms.

## AUTHOR CONTRIBUTIONS

**José M. Izquierdo:** Conceptualization, visualization, writing—original draft preparation, writing—reviewing, funding acquisition and editing.

## CONFLICT OF INTEREST STATEMENT

The author has no competing interests to declare.

## Data Availability

Not Applicable.

## References

[acel13927-bib-0001] Bentley, D. L. (2014). Coupling mRNA processing with transcription in time and space. Nature Reviews. Genetics, 15, 163–175. 10.1038/nrg3662 PMC4304646 24514444

[acel13927-bib-0002] Chen, Y. , Chafin, D. , Price, D. H. , & Greenleaf, A. L. (1996). *Drosophila* RNA polymerase II mutants that affect transcription elongation. The Journal of Biological Chemistry, 271, 5993–5999. 8626382

[acel13927-bib-0003] Cylus, J. , Roubal, T. , Ong, P. , & Barber, S. (2019). European observatory policy briefs. In A. Sagan , C. Normand , J. Figueras , J. North , & C. White (Eds.), Sustainable health financing with an ageing population: Implications of different revenue raising mechanisms and policy options. Copenhagen European Observatory on Health Systems and Policies. World Health Organization. 31820888

[acel13927-bib-0004] Debès, C. , Papadakis, A. , Grönke, S. , Karalay, O. , Tain, L. S. , Mizi, A. , Nakamura, S. , Hahn, O. , Weigelt, C. , Josipovic, N. , Zirkel, A. , Brusius, I. , Sofiadis, K. , Lamprousi, M. , Lu, Y. X. , Huang, W. , Esmaillie, R. , Kubacki, T. , Späth, M. R. , … & Beyer, A. (2023). Ageing‐associated changes in transcriptional elongation influence longevity. Nature, 616, 814–821. 37046086 10.1038/s41586-023-05922-y PMC10132977

[acel13927-bib-0005] Feser, J. , Truong, D. , Das, C. , Carson, J. J. , Kieft, J. , Harkness, T. , Jessica, K. , & Tyler, J. K. (2010). Elevated histone expression promotes lifespan extension. Molecular Cell, 39, 724–735. 20832724 10.1016/j.molcel.2010.08.015 PMC3966075

[acel13927-bib-0006] Li, Y. I. , Knowles, D. A. , Humphrey, J. , Barbeira, A. N. , Dickinson, S. P. , Im, H. K. , & Pritchard, J. K. (2018). Annotation‐free quantification of RNA splicing using LeafCutter. Nature Genetics, 50, 151–158. 29229983 10.1038/s41588-017-0004-9 PMC5742080

[acel13927-bib-0007] López‐Otín, C. , Blasco, M. A. , Partridge, L. , Serrano, M. , & Kroemer, G. (2023). Hallmarks of aging: An expanding universe. Cell, 186, 243–278. 36599349 10.1016/j.cell.2022.11.001

[acel13927-bib-0008] Lu, T. C. , Brbić, M. , Park, Y. J. , Jackson, T. , Chen, J. , Kolluru, S. S. , Gi, Y. , Katheder, N. S. , Cai, X. T. , Lee, S. , Chen, Y. C. , Auld, N. , Liang, C. Y. , Ding, S. H. , Welsch, D. , D'Souza, S. , Pisco A. O. , Jones, R. C. , Leskovec, J. , … Li, H. (2023). Aging fly cell atlas identifies exhaustive aging features at cellular resolution. Science, 380, eadg0934. 37319212 10.1126/science.adg0934 PMC10829769

[acel13927-bib-0009] Lu, Y. X. , Regan, J. C. , Eßer, J. , Drews, L. F. , Weinseis, T. , Stinn, J. , Hahn, O. , Miller, R. A. , Grönke, S. , & Partridge, L. (2021). A TORC1‐histone axis regulates chromatin organization and non‐canonical induction of autophagy to ameliorate ageing. eLife, 10, e62233. 33988501 10.7554/eLife.62233 PMC8186904

[acel13927-bib-0010] Martin, R. D. , Hébert, T. E. , & Tanny, J. C. (2020). Therapeutic targeting of the general RNA polymerase II transcription machinery. International Journal of Molecular Sciences, 21, 3354. 32397434 10.3390/ijms21093354 PMC7246882

[acel13927-bib-0011] Mimoso, C. A. , & Adelman, K. (2023). U1 snRNP increases RNA pol II elongation rate to enable synthesis of long genes. Molecular Cell, 83, 1264–1279. 36965480 10.1016/j.molcel.2023.03.002 PMC10135401

[acel13927-bib-0012] Muniz, L. , Nicolas, E. , & Trouche, D. (2021). RNA polymerase II speed: A key player in controlling and adapting transcriptome composition. The EMBO Journal, 40, e105740. 34254686 10.15252/embj.2020105740 PMC8327950

[acel13927-bib-0013] Naftelberg, S. , Schor, I. E. , Ast, G. , & Kornblihtt, A. R. (2015). Regulation of alternative splicing through coupling with transcription and chromatin structure. Annual Review of Biochemistry, 84, 165–198. 10.1146/annurev-biochem-060614-034242 26034889

[acel13927-bib-0014] Oberdoerffer, P. (2010). An age of fewer histones. Nature Cell Biology, 12, 1029–1031. 21045802 10.1038/ncb1110-1029

[acel13927-bib-0015] Pan, Y. H. , Wu, W. P. , & Xiong, X. D. (2020). Circular RNAs: Promising biomarkers for age‐related diseases. Aging and Disease, 11, 1585–1593. 33269108 10.14336/AD.2020.0309 PMC7673852

[acel13927-bib-0016] Panigrahi, A. , & O'Malley, B. O. (2021). Mechanisms of enhancer action: The known and the unknown. Genome Biology, 22, 108. 33858480 10.1186/s13059-021-02322-1 PMC8051032

[acel13927-bib-0017] Rogalska, M. E. , Vivori, C. , & Valcárcel, J. (2023). Regulation of pre‐mRNA splicing: Roles in physiology and disease, and therapeutic prospects. Nature Reviews. Genetics, 24, 251–269. 10.1038/s41576-022-00556-8 36526860

[acel13927-bib-0018] Rogalski, T. M. , Bullerjahn, A. M. , & Riddle, D. L. (1988). Lethal and amanitin‐resistance mutations in the *Caenorhabditis elegans* ama‐1 and ama‐2 genes. Genetics, 120, 409–422. 3197954 10.1093/genetics/120.2.409 PMC1203520

[acel13927-bib-0019] Selman, C. , Lingard, S. , Choudhury, A. I. , Batterham, R. L. , Claret, M. , Clements, M. , Ramadani, F. , Okkenhaug, K. , Schuster, E. , Blanc, E. , Piper, M. D. , Al‐Qassab, H. , Speakman, J. R. , Carmignac, D. , Robinson, I. C. A. , Thornton, J. M. , Gems, D. , Partridge, L. , & Withers, D. J. (2008). Evidence for lifespan extension and delayed age‐related biomarkers in insulin receptor substrate 1 null mice. The FASEB Journal, 22, 807–818. 17928362 10.1096/fj.07-9261com

[acel13927-bib-0020] Singh, P. , Gollapalli, K. , Mangiola, S. , Schranner, D. , Yusuf, M. A. , Chamoli, M. , Shi, S. L. , Bastos, B. L. , Nair, Y. , Riermeier, A. , Vayndorf, E. M. , Wu, J. Z. , Nilakhe, A. , Nguyen, C. Q. , Muir, M. , Kiflezghi, M. G. , Foulger, A. , Junker, A. , Devine, J. , … Yadav, V. K. (2023). Taurine deficiency as a driver of aging. Science, 380, eabn9257. 37289866 10.1126/science.abn9257 PMC10630957

[acel13927-bib-0021] Soares, L. M. , He, P. C. , Chun, Y. , Suh, H. , Kim, T. , & Buratowski, S. (2017). Determinants of histone H3K4 methylation patterns. Molecular Cell, 68, 773–785.e776. 29129639 10.1016/j.molcel.2017.10.013 PMC5706784

[acel13927-bib-0022] Sural, S. , Liang, C. Y. , Wang, F. Y. , Ching, T. T. , & Hsu, A. L. (2020). HSB‐1/HSF‐1 pathway modulates histone H4 in mitochondria to control mtDNA transcription and longevity. Science Advances, 6, eaaz4452. 33087356 10.1126/sciadv.aaz4452 PMC7577724

[acel13927-bib-0023] Tilgner, H. , Nikolaou, C. , Althammer, S. , Sammeth, M. , Beato, M. , Valcárcel, J. , & Guigo, R. (2009). Nucleosome positioning as a determinant of exon recognition. Nature Structural & Molecular Biology, 16, 996–1001. 10.1038/nsmb.1658 19684599

[acel13927-bib-0024] Tyshkovskiy, A. , Ma, S. , Shindyapina, A. V. , Tikhonov, S. , Lee, S. G. , Bozaykut, P. , Castro, J. P. , Seluanov, A. , Schork, N. J. , Gorbunova, V. , Dmitriev, S. E. , Miller, R. A. , & Gladyshev, V. N. (2023). Distinct longevity mechanisms across and within species and their association with aging. Cell, 186, 2929–2949.e20. 37269831 10.1016/j.cell.2023.05.002 PMC11192172

[acel13927-bib-0025] Wouters, J. , Kalender Atak, Z. , & Aerts, S. (2017). Decoding transcriptional states in cancer. Current Opinion in Genetics & Development, 43, 82–92. 28129557 10.1016/j.gde.2017.01.003

[acel13927-bib-0026] Zhang, W. W. , Qu, J. , Liu, G. H. , & Izpisúa‐Belmonte, J. C. (2020). The ageing epigenome and its rejuvenation. Nature Reviews. Molecular Cell Biology, 21, 137–150. 32020082 10.1038/s41580-019-0204-5

[acel13927-bib-0027] Zhang, Y. , Xue, W. , Li, X. , Zhan, J. , Chen, S. , Zhang, J. L. , & Yang, L. (2016). The biogenesis of nascent circular RNAs. Cell Reports, 15, 611–624. 27068474 10.1016/j.celrep.2016.03.058

